# Atypical Presentation of Ludwig Angina: Diagnostic and Therapeutic Complications in a Rare Case

**DOI:** 10.7759/cureus.98046

**Published:** 2025-11-28

**Authors:** Himaja Movva, Genevieve A Crawley, Vy Nguyen, Peyman Arghavani

**Affiliations:** 1 Internal Medicine, Ross University School of Medicine, Miramar, USA; 2 Internal Medicine, Dignity Health Hospital California Medical Center, Los Angeles, USA; 3 Medical Oncology, Internal Medicine, and Surgery, Ross University School of Medicine, Miramar, USA

**Keywords:** acute pain, dental hygiene education, facial swelling, floor of mouth cellulitis, general internal medicine, infectious disease medicine, ludwig's angina, mouth diseases, parotid lymph node, submandibular glands

## Abstract

Finding the clinical significance of facial swelling was of utmost importance in this case. Ludwig angina is a life-threatening cellulitis of the floor of the mouth that is mainly due to infectious causes. This particular patient had an atypical presentation except for the facial swelling in which the patient’s non-specific symptoms made it difficult to obtain a diagnosis. Severe edema can make it difficult to intubate the patient; however, our case did not present with any common symptoms such as this. This is why it is of utmost importance to maintain dental/mouth hygiene and maintenance because of complications such as Ludwig angina. Ludwig angina is becoming more common amongst hospitals, but being able to obtain a diagnosis quickly even with atypical symptoms is of top priority for resolution.

## Introduction

Ludwig angina is a life-threatening cellulitis of the soft tissue involving the floor of the mouth and neck. The condition was named after a German physician, Wilhelm Friedrich von Ludwig, who described it in 1836 [1]. Ludwig angina involves three compartments of the floor of the mouth: sublingual, submental, and submandibular. It mainly originates from dental infections in the mandibular molars, particularly periapical abscesses in the second or third mandibular molars, which account for over 90% of cases [2,3]. The disease is usually polymicrobial and involves oral flora, both aerobes and anaerobes. Often arising from odontogenic infections and typically seen in patients with systemic risk factors such as diabetes or immunosuppression, the most common organisms are Staphylococcus, Streptococcus, Peptostreptococcus, Fusobacterium, Bacteroides, and Actinomyces [2,4]. Prompt recognition and intervention are critical to prevent airway compromise, septic progression, and mortality. Ludwig angina is often characterized by a distinctive physical appearance referred to as a "bull neck" [2]. Patients frequently present with recent dental pain, systemic symptoms including fever, fatigue, chills, and overall weakness. Additional signs and symptoms may involve oral pain, voice changes, drooling, tongue swelling, elevation of the mouth floor, and neck stiffness [5]. Upon examination, patients commonly exhibit fever along with swelling and tenderness in the submental and submandibular regions. Intraoral signs often include floor-of-mouth swelling, trismus, tongue elevation, and tenderness of the involved teeth. Extraoral findings may involve induration of the submental area and edema of the upper neck [2]. Other complications include mediastinitis, carotid artery rupture, internal jugular vein thrombophlebitis, empyema, necrotizing fasciitis, osteomyelitis, and aspiration pneumonia [6,7]. Airway management is the most critical factor influencing survival in patients with Ludwig’s angina. Once the airway is secured, treatment shifts to controlling the infection using intravenous broad-spectrum antibiotics and, when necessary, surgical drainage - often including extraction of the affected teeth. Intravenous corticosteroids and nebulized epinephrine may be used as adjunct therapies to help reduce facial and airway swelling [2].

This case is unique because it involves an immunocompetent male with no past medical history or identifiable risk factors, who presented with right facial swelling and odynophagia without systemic symptoms or preceding trauma. The instructive value of this case lies in its atypical presentation and progression with absence of fever, drooling, or respiratory distress. In the absence of systemic comorbidities, the patient developed a significant deep neck space abscess requiring operative drainage and aggressive antibiotic therapy. This emphasizes the need for clinicians to maintain a high index of suspicion for Ludwig’s angina, even in healthy individuals without classic risk factors, and to recognize that the absence of classic symptoms does not exclude severe underlying infection. Furthermore, the case demonstrates the effectiveness of multidisciplinary management with ENT, infectious disease, and critical care teams, reinforcing a cost-effective, systematic approach to prevent airway compromise and promote recovery in cases of deep neck infections.

## Case presentation

The patient was a 63-year-old male with no past medical history who initially presented to the hospital with complaints of 7/10 constant burning right facial pain and swelling. He reported that the swelling started three days ago and does not remember any inciting trauma and/or bite. The patient denies swelling in this location before. He described difficulty swallowing due to the inflammation, not the pain itself, and the pain was exacerbated by chewing. He denied fever, dyspnea, chest pain, drooling, or xerostomia.

The patient denied any past medical history or past surgery history, and his family history was noncontributory. The patient denied taking any medications, tobacco use, alcohol consumption, or illicit drug use. There are no known drug allergies recorded. The vital signs were monitored and within normal limits. 

During the physical examination, the patient was in no acute distress and was lying comfortably in bed. Lung auscultation was clear, with no crackles or wheezes. Cardiovascular examination revealed a regular rate and rhythm, normal S1 and S2 heart sounds, and no murmurs. Neurologically, he was alert and oriented to person, place, and time. Cranial nerves II-XII were intact, and no focal neurological deficits were noted.

**Figure 1:**
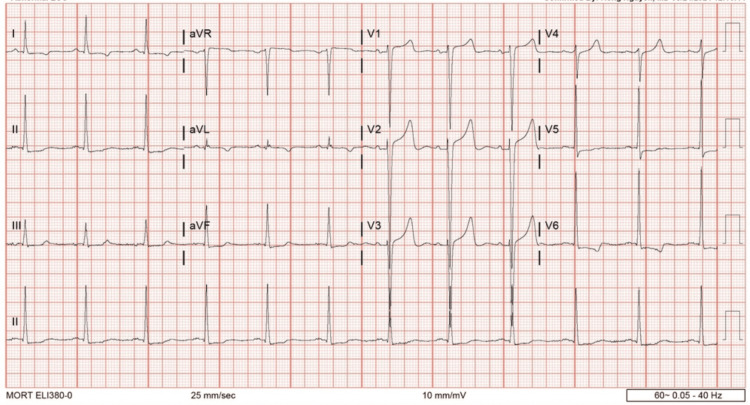
EKG of Ludwig angina patient

**Figure 2:**
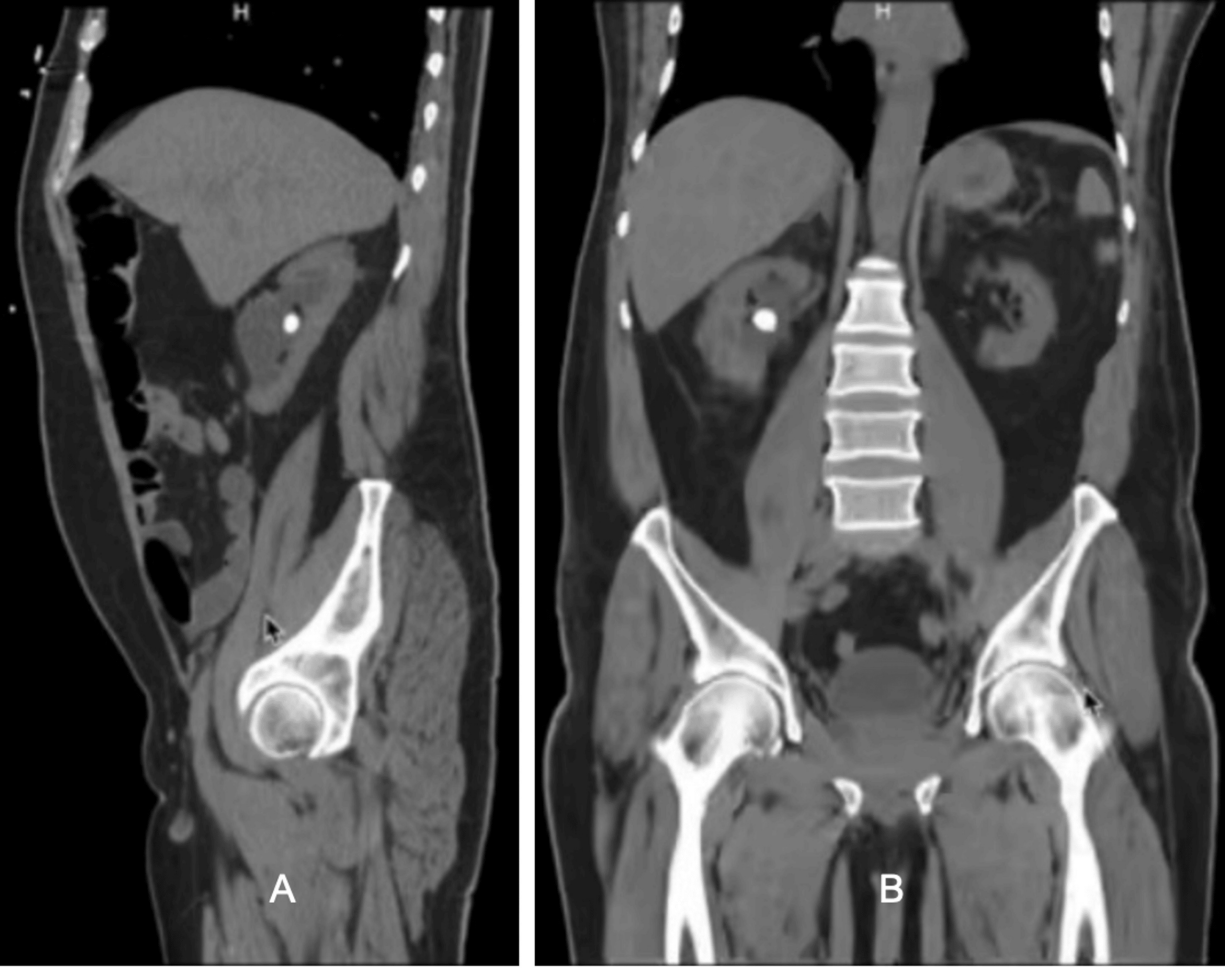
A- Sagittal view; B- Coronal view. Both panels A and B show CT abdomen + pelvis with non-acute urologic finding of probable kidney stone.

Labs were taken on admission revealed elevated white blood cell at 27.7 (4500-11,000/mm3), procalcitonin level at 1.77 (<0.05 ng/mL), troponin at 70.3 (<0.04 ng/dL), B-type natriuretic peptide (BNP) at 854.4 (<100 pg/mL), blood urea nitrogen (BUN) at 51 (7-18 mg/dL), creatinine level at 7.4 (0.6-1.2 mg/dL), with decreased estimated glomerular filtration rate (eGFR) at 8 and normal anion gap and lactic acid. Cardiology and Urology were consulted to follow up. EKG was performed and was significant for left ventricular hypertrophy and ST-T changes shown in Figure 1. CT abdomen/pelvis presented a non-acute urologic finding of probable kidney stone which is shown in Figures 2A, 2B. The patient was then recommended to follow up outpatient for further care and observation. 

**Figure 3:**
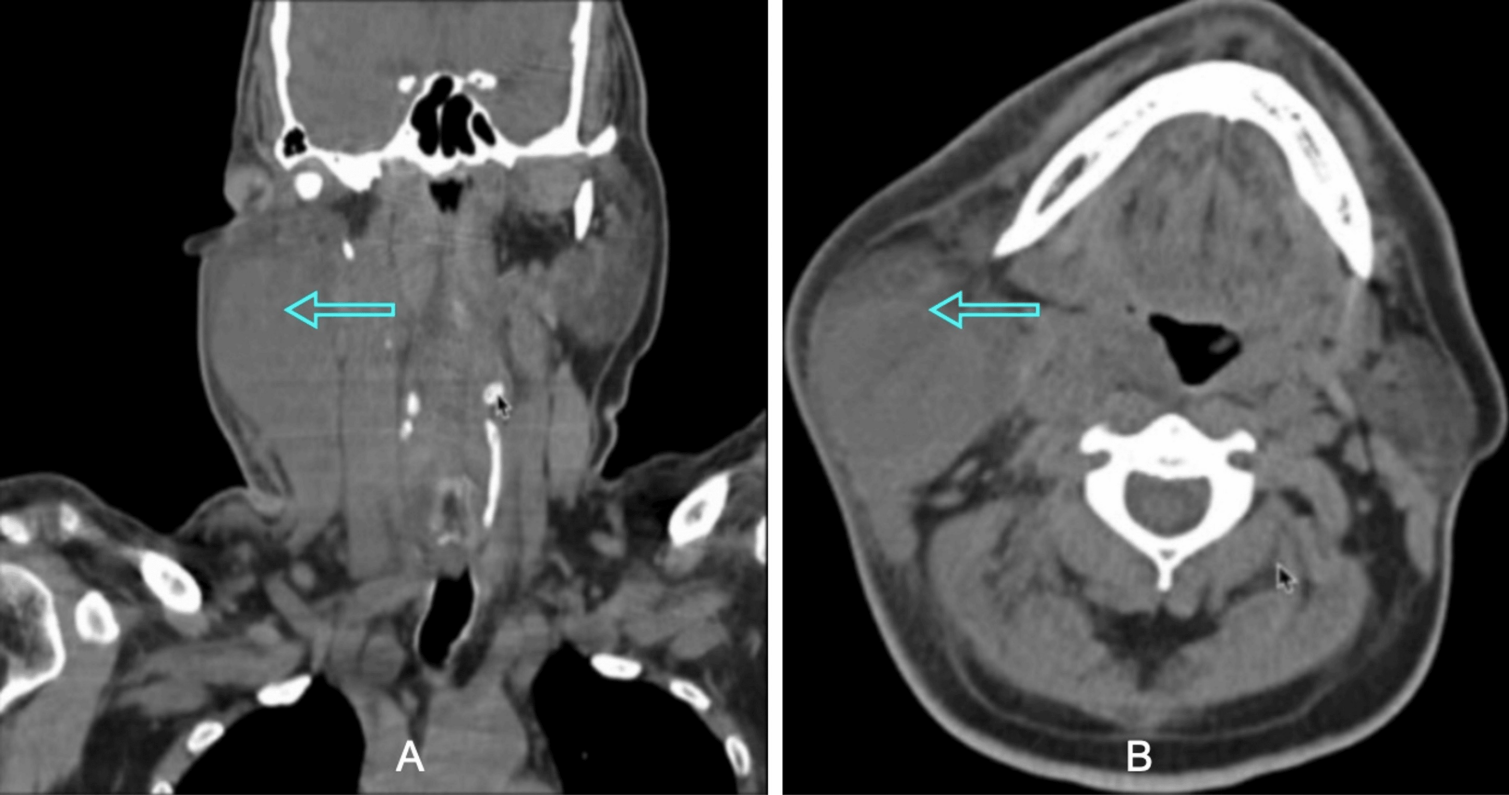
A- Coronal View; B- Axial View. Both panels A and B show CT neck soft tissue without contrast showing right-sided Ludwig angina.

CT neck was performed and notable for approximately 5.2 cm AP x 4.9 cm TR x 6.0 cm soft tissue lesion along the mid/lower lateral face, which could have been from the inferior aspect of the superficial lobe of the right parotid gland. There was mild to moderate diffuse enlargement of the right submandibular gland and moderate diffuse soft tissue edema and mild swelling of the right lower face and submental region, right greater than left. Several right peri-parotid, right Level II/IB, and left Level II/IB non-necrotic lymph nodes measuring up to 1.5 cm in short axis and 1.7 cm in long axis. CT of the soft tissue of the neck without contrast is shown below in Figure 3A, 3B.

Blood culture was ordered and came back negative. The patient was given clindamycin, vancomycin and Decadron 10 mg in the emergency room, which later continued with clindamycin and cefepime, and was placed on IV fluid and nothing by mouth (NPO) when admitted to the floor. ENT was consulted and scheduled for incision and drainage of right deep neck abscess. Intraoperatively, a large fluctuant mass was found below the mandible, inferior to the tail of the parotid. An obvious malodorous abscess cavity was encountered in which a culture was taken. The wound was copiously irrigated with diluted Betadine solution. A 1/2-inch Penrose drain was placed in the wound and the wound was closed in layers with interrupted 3-0 chromic suture. Through the physical exam findings, clinical signs and symptoms as previously described, and confirmatory CT scans the patient was diagnosed with Ludwig angina. The patient was aroused and extubated and transferred to the recovery room in satisfactory condition. The patient recovered and had a full understanding of his condition. Culture of the abscess came back positive for Salmonella and Salmonella Typhi, which prompted the doctor to prescribe a 14-day course of oral cefdinir antibiotic. He was discharged home five days after his initial presentation to the emergency department.

## Discussion

This case highlights facial swelling complications and clinical significance in adult patients, particularly when presenting with atypical symptoms. This case further supports that a patient’s facial swelling symptoms should not go unnoticed, as there is a possibility for a more severe pathology. The patient’s atypical presentation without respiratory compromise and sepsis is what makes this case unique and difficult to diagnose. The patient’s physical exam showed that he was in no acute respiratory distress and normal throughout all organ systems, except for right facial swelling. A probable diagnosis of right facial swelling is a peritonsillar abscess, which can spread to cause Ludwig's angina; however, the alarming symptoms of trismus, "hot potato voice", or drooling [8] were not clinically observed in this patient. Not having these symptoms led us to use other pieces of information such as the patient's labs in order to help guide diagnosis. In accordance with the patient’s labs, he had leukocytosis and negative blood cultures, which was unusual given the size of his facial swelling. A majority of Ludwig angina cases are dental hygiene related, while others include immunosuppression, diabetes, obesity, and non-steroidal anti-inflammatory drug (NSAID) use amongst others. Another unique aspect in this patient’s case history is his lack of risk factors or unknown source of anaerobic infection. It is imperative for patient history to be meticulously recorded because this patient wasn’t specifically questioned regarding possible NSAID use. If NSAID use is also ruled out, then this patient didn’t present with any of the usual risk factors, which should be further investigated. A source of infection was unable to be identified due to negative blood cultures, which also poses another complication in this case. The treatments for Ludwig angina are antibiotics, incision and drainage, pain management, and tooth extraction if due to tooth complication [9]. The preferred antibiotic therapy involves coverage for broad-spectrum with β-lactams, often in combination with clindamycin for polymicrobials, which must be synergistically combined with nutritional support and yearly dental examinations. Mortality from Ludwig angina has been reported to exceed 50%; however, it substantially decreases with the use of the above combination of medical therapies [10].

## Conclusions

This case highlights the challenges of facial swelling in an older male with no present risk factors and minimal systemic symptoms. This patient’s form of Ludwig angina had an insidious onset that caused him to have an atypical presentation along with facial swelling. This challenges the prevailing understanding that Ludwig’s angina primarily affects individuals with systemic risk factors and demonstrates the need for high clinical suspicion and prompt imaging, even in immunocompetent patients presenting with localized symptoms. Prompt CT imaging or US at ED (reducing CT costs) should be reinforced even if physical exam findings are unsuspicious. This is why it is of utmost importance to be aware of the clinical symptoms of Ludwig angina so that when there are little to no symptoms we are still able to obtain a clinical diagnosis quickly and efficiently. Maintaining clean dental and mouth hygiene practices will also help to decrease the incidence of Ludwig angina.
